# On the Treatment of Prolapsus Ani

**Published:** 1848-05

**Authors:** 


*On the treatment of Prolapsus Ani.* By MR. VINCENT.—"Of late I have found such great advantage in employing the sulphate of iron in prolapsed bowel, that the operation may very often be dispensed with, and the patient quite cured merely with the use of this remedy. Very lately I had in the hospital two cases of the worst sort,—the one of twenty years' standing, with a great protrusion and abundance of bleeding piles, who in about three weeks left without any protrusion or bleeding; declaring himself to be in a state of comfort that he had not known for so long a time. The other came from one of the institutions that offer great pretensions in the treatment of this class of cases. He was very bad, having both internal and external piles, and the bowel descending largely and most readily; he was completely relieved in about a month. Other cases of a slighter kind have been set to rights in not much more than a week. The patient should be kept in bed of course, so that there should be every facility for the repose of the bowel; and after it is cleansed out, a small quantity of the injection should be daily thrown up, and retained. If the stomach can take balsams, they seem well adapted for this disease."—*Ibid, from Observations on some parts of Surgical Practice.*

